# An Integrative Approach to Ketamine Therapy May Enhance Multiple Dimensions of Efficacy: Improving Therapeutic Outcomes With Treatment Resistant Depression

**DOI:** 10.3389/fpsyt.2021.710338

**Published:** 2021-11-24

**Authors:** Sherry-Anne Muscat, Glenn Hartelius, Courtenay Richards Crouch, Kevin W. Morin

**Affiliations:** ^1^Youth Forensic Psychiatry, Alberta Hospital, Alberta Health Services, Edmonton, AB, Canada; ^2^Integral and Transpersonal Psychology, California Institute of Integral Studies, San Francisco, CA, United States; ^3^Department of Psychiatry, University of Alberta, Edmonton, AB, Canada; ^4^Acute Adult Psychiatry, Alberta Hospital, Alberta Health Services, Edmonton, AB, Canada

**Keywords:** integrative ketamine therapy, treatment resistant depression, NMDAR modulator, normalized brain connectivity, induced neural plasticity, anti-inflammatory, entropic brain states, psychedelic peak experience

## Abstract

Research over the last two decades has established ketamine as a safe, effective, fast-acting, and sustained antidepressant that significantly reduces adverse symptoms associated with depression, even in patients who are treatment resistant. Much of this research has evolved within the framework of several independent branches of scientific inquiry: in addition to the study of ketamine is a non-selective NMDAR antagonist with rapid antidepressant effects, it has also been found effective as a psychoplastogen that stimulates synaptogenesis and increases neuroplasticity, as a powerful anti-inflammatory that may improve inflammation-related depressive symptoms, as a substance that induces beneficial high entropy brain states, and as a subjectively impactful psychedelic agent. Each branch of inquiry has generated independent evidence of ketamine's efficacy but has advanced without substantive coordination or communication with other lines of inquiry. Integrative research that considers these branches of research together may lead toward a better understanding of ketamine's effects and improved treatment protocols and clinical outcomes. Such an overview can inform more comprehensive patient care through: (a) informed patient psychoeducation that encompasses all of ketamine's mechanisms of action; (b) calibration of optimal dosage to ensure induction and maintenance of high entropy brain states during each ketamine session utilizing EEG measurement; (c) Improved management of emergence side effects through proper care for set and setting; (d) inclusion of pre-selected appropriate music to enhance the emotional experience; (e) increased monitoring of ketamine effects on cortical activity, inter-hemispheric imbalance, and inflammation-related levels of cytokines to further improvements in ketamine protocols; and (f) appropriate timing of any adjunctive psychotherapy sessions to coincide with peak neurogenesis at 24–48 h post ketamine treatment.

## Introduction and Background

The World Health Organization (1) has declared depression a global epidemic, confirming that over 300 million people suffer from major depressive disorder (MDD) worldwide, a number is increasing exponentially. As a major contributor to the overall global burden of disease, MDD has been identified as the leading cause of disability. In addition, annually approximately 800,000 people take their own life. Statistically, the incidence rate of MDD in the USA is 6.4% of the adult population, of which treatment resistant MMD (TRD) accounts for ~12–20% of that population (2). Unfortunately, the current prognosis for recovery (≥50% depression symptom reduction) from MMD is poor. Clinical trials have consistently revealed that the effectiveness of traditional antidepressants of all classes over and above placebo is small and falls well-below the recommended criteria for clinical effectiveness (3). Furthermore, in the cases where conventional antidepressants are effective, it often takes months for a positive response. Additionally, only 50% of those patients go into remission, and depression symptoms frequently return (4).

Littman and Krishna (5) maintained that even with problematic efficacy statistics, the discovery of serotonin selective reuptake inhibitors (SSRIs) in the late 1980s and early 1990s was a fundamental step in advancing the treatment of depression and other mental disorders. The development of SSRIs introduced the key concept of designing psychotropic medications that both targeted specific biological structures and networks in the brain while limiting the effect on non-targeted biological structures, thus decreasing the adverse side effects that plagued earlier psychotropic medications. Typical antidepressants that target monoamines are only moderately effective in blocking or reversing these neuronal structural changes and require chronic administration to modulate synaptic activity.

The alarming accumulative statistics of depression and its debilitating impact on society, combined with the modest treatment efficacy of standard antidepressants, prompted the study of traditional and non-traditional psychedelics such as ketamine as a novel treatment modality for depression and other mental disorders. Initial research findings have been encouraging, and the response from the medical community cautiously optimistic, resulting in widespread research activity (6). Today, psychedelics have become a research focus at major universities such as Harvard, Johns Hopkins, and Imperial College London (7–9).

In the past two decades, ketamine, a non-competitive N-methyl-D-aspartate (NMDA) receptor antagonist, has shown promise as a novel treatment modality for TRD. Although not considered a traditional serotonergic psychedelic, ketamine is currently the only legally obtainable drug with psychedelic effects and is therefore more readily accessible for research and clinical use. Ketamine has been used for more than 50 years as an effective short-acting anesthetic for all ages, has an excellent and well-documented safety record, and does not normally interfere with respiration, cardiovascular stability, or airway collapse even at maximum effect. Ketamine has been touted as a wonder drug for emergency medicine worldwide as it is easy to administer even in combat conditions, has potent analgesic and anti-inflammatory effects, is appropriate for almost any age, and is short-acting with few known side effects. In recent years physicians experienced with ketamine's effects and confident in its safety profile and legal status have incorporated the off-label prescription of ketamine for mental disorders into their clinical practice (10–12).

A key factor in ketamine's efficacy with mental disorders is its role as a powerful and efficient glutamate receptor modulator—the only known psychedelic substance with this effect. Glutamate is the main excitatory neurotransmitter in the mammalian central nervous system, is present in over 60% of all brain synapses and is the primary mediator of neurosystem plasticity (13). Glutamate levels are tightly regulated by homeostatic excitatory and inhibitory neurotransmission through a complex system of ionotropic and metabotropic receptors throughout the whole neuraxis. Any abnormality in glutamate function or in the regulation of glutamatergic transmission can disrupt nerve health and communication and is a major influence on the presentation and progression of many neurodegenerative and psychiatric diseases (14–18).

Although ketamine has been investigated and utilized extensively for a variety of its properties, including newly discovered neuroprotective, anti-inflammatory, and anti-tumor effects (14, 15), it continues to be a controversial substance due to what is termed ketamine emergence side effects, reportedly experienced by 30–40% of patients post anesthesia (11). Emergence phenomena are described as being in a dissociative state or as having an out of body experience. There may be vivid imagery, a feeling of loss of reality, hallucinations, overexcitement, and behavior that appears irrational to others (11). Emergence phenomena are also experienced at subanesthetic doses as low as 0.5 mg/kg. Other side effects at subanesthetic doses include memory recall and recognition, explicit and implicit memory impairment, and decreases in mental sharpness and concentration during or shortly after administration. Some individuals may experience increased agitation or anxiety/panic attacks (12). To control or suppress these effects doctors regularly administer benzodiazepines or more recently propofol to control or suppress the emergent side effects post anesthesia (14). In addition, ketamine has become popular for recreational use due to its hallucinogenic and dissociative effects.

While dependency and overuse can and does occur in unsupervised settings, ketamine is not known or classified as a highly addictive drug (11, 12, 15, 17). The efficacy of ketamine for major depressive disorder, post-traumatic stress disorder, chronic pain, end of life resolution, alcoholism, drug addictions, and other psychological problems is well-documented in the literature (7, 16, 19–23). Over the last two decades research has demonstrated ketamine's effectiveness in the treatment of MDD and the rapid resolution (4 h) of suicidal ideation, an acute symptom associated with MDD (12, 24–27).

As research into ketamine treatment for MDD has progressed, several distinct branches of inquiry into its effects have emerged with different and often conflicting motivations and objectives, with minimal integration of findings. One branch of inquiry has focused on ketamine's antidepressant effects as a bi-hemispherical homeostatic glutamate receptor modulator, both as inhibitor and excitor; another has explored ketamine's effects on chronic stress-induced inflammation; a third has concentrated on the discovery that ketamine stimulates synaptogenesis and dendritic spine growth inherent in neuronal communication and connectivity; a fourth has considered the neurological impact of the psychedelic state associated with ketamine at subanesthetic dosages; and a 5th focus has been on the phenomenology of the ketamine experience and its subjective impact. There is also interest in the efficacy of ketamine as an adjunct to psychotherapy. However, protocols for subanesthetic ketamine treatment and ketamine assisted psychotherapy developed to date (11) have not been optimized to account for the drug's multiple properties (12).

## Hypothesis

Integrating the data on ketamine's five primary characteristic actions—N-methyl-D-aspartate receptor (NMDAR) modulation, neuroplastogenic effects, anti-inflammatory effects, entropic brain state modulation, and psychedelic induction of peak or mystical experiences—may improve treatment protocols and clinical outcomes by guiding educational preparation of patients, optimization strategies for ketamine dosing and dose regulation, informing management of dissociative emergence effects, monitoring impacts on brain activity, and predicting favorable timing for adjunct psychosocial interventions.

## Therapeutic Effects of Ketamine

As a biochemical agent, ketamine is a glutamate receptor modulator, and when administered, has sustained, fast acting antidepressant effects on many individuals diagnosed with TRD. There is evidence that ketamine selectively induces synaptogenesis and neuroplasticity in brain structures and systems affected by depression-related neuronal atrophy, thereby potentially initiating the development of neurological architecture supportive of adaptive experience related neural pathways, while aiding the therapeutic disruption of entrenched patterns of neural activation associated with negative thoughts and beliefs (28, 29). Ketamine accomplishes these enhancements as an NMDA receptor antagonist that increases presynaptic glutamate release, increases AMPA activities associated with the regulation of synaptic function, and suppresses NMDA receptor activities (30). Carhart-Harris et al. (31) have postulated that psychedelic substances induce a high entropy brain state, which may be instrumental in ketamine's efficacy with TRD patients. The experience of ketamine's psychoactive effects also offers some individuals with TRD reconnection with, or even discovery of, an experience that has been described as a meaningful, transcendent experience of oneness, connectivity, hope, and happiness (19, 23, 32–35). These subjective effects of ketamine treatment may enhance the ability to disidentify with rigid negative core self-beliefs.

### Ketamine as NMDAR Modulator

Ketamine is classified as a non-competitive ionotropic glutamatergic N-methyl-D-aspartate receptor (NMDAR) antagonist (35). Ketamine's direct mechanisms of rapid and prolonged antidepressant action appears to be a synergetic interplay of the inhibition of GABAergic interneuron NMDARs and resulting increase of glutamate excitability in the medial prefrontal cortex (mPFC) and overall cortical disinhibition, and the downstream systems involved in the regulation of entropy, anti-inflammatory properties, and neural plasticity. The rapid actions of ketamine are in part mediated via regulation of glutamate ionotropic receptors including the α-amino-3-hydroxy-5-methyl-4-isoxazolepropionic acid (AMPA) and NMDA subtypes, which are required for cellular and behavioral models of short- and long-term learning and memory, including regulation of the number and function of spine synapses.

However, ketamine is a selective NMDAR antagonist. In addition to its role as an essential glutamate receptor modulator, ketamine appears to target and inhibit hyperpolarization-activated cyclic nucleotide-gated (HCN) channels in the ventral tegmental area (VTA), the prefrontal cortex (PFC), and the hippocampus (36). In rodent tissue samples ketamine inhibits I_*h*_—the membrane current mediated by HCN channels—by inducing membrane hyperpolarization. Moreover, antidepressant effects of ketamine are suppressed in HCN knockout mice (37, 38). Similarly, in post chronic stress exposure rodents, but not acute stress exposure rodents, the I_*h*_ and HCN1 protein expression were increased in the dorsal, but not ventral, CA1 region (39, 40). This upregulation contributed to a reduction of neuronal excitability similar to depressed patients, which was attenuated by ketamine treatment. Furthermore, this study found that the reduced HCN1 protein expression in dorsal CA1 region resulting from post chronic stress ketamine treatment produced subsequent resiliency effects on re-exposure to chronic stress. Increases in HCN1 protein expression are thus implicated in the development of depression and a treatment-related reduction of HCN1 protein expression is associated with resilience to later chronic stress exposure (39, 40). Ketamine's ability to target these ion channels deserves additional consideration as one of its likely antidepressant mechanisms of action, illustrating the fact that the interplay of known and as-yet unknown variables contributing to the overall efficacy of ketamine is still under investigation (11, 35, 41).

Berman et al. (42) conducted the first human clinical trial of ketamine for treatment resistant patients with major depressive disorder (MDD) with a small yet diverse (across age, ethnicity, and sex), subject sample size; although the study was of limited scope, results were encouraging. The researchers employed a randomized, double-blind crossover design in which each of seven adult participants with confirmed DSM-IV diagnosis of treatment resistant major depressive disorder received one trial of a single intravenous 0.5 mg/kg dose of ketamine hydrochloride solution over 40 min duration, and one trial of a single intravenous dose of placebo saline, with a minimum one-week interval between trials. The study administered several psychometric measures at baseline including the Hamilton Depression Rating Scale (HDRS), the Visual Analog Scales high mood scale, the Beck Depression Inventory, and the Brief Psychiatric Rating Scale; each measure was readministered four to five times during the 6 h post infusion, and the HDRS was given at 72 h after ketamine treatment.

Berman et al.'s (42) results supported the hypothesis that a single dose of ketamine significantly reduced depression symptoms in treatment resistant subjects. Results also illustrated ketamine's rapid and sustained antidepressant effects, with significant symptom reduction onset within four h of ketamine infusion, and peak symptom reduction at 72 h post infusion. Four of the 7 subjects demonstrated a depression symptoms reduction of > 50% above placebo at 72 h post infusion with positive effects that lasted longer than 72 h. Subjects reported that their ketamine-induced intoxication / high dissipated to baseline within a few h post infusion. Beyond an affective elevation of mood ketamine reduced the depressive symptoms of suicidality, helplessness, and hopelessness, driving the conclusion that ketamine has true antidepressant qualities beyond the effects that could be attributed to a ketamine-induced intoxication/ euphoria state (35, 42).

Berman et al. (42) reported that their data was consistent with the previous animal models that demonstrated the rapid and sustained antidepressant qualities of ketamine on depression/stress symptoms. Additionally, the data confirmed the likely role of glutamate in mood disorders. However, Berman et al. strongly advised that future research should focus on the synthesis of an NMDA receptor antagonist drug that minimized or eliminated the adverse psychotomimetic effects of ketamine, warning that the dissociative, euphoric quality of ketamine, and the potential for ketamine abuse, would severely hamper the usefulness of ketamine in clinical settings. This cautionary sentiment set the tone and influenced the pathway for much of the extensive subsequent research into the use of ketamine for TRD.

Prompted by ketamine's potential to mitigate the suffering, acute suicidality, and mortality associated with the delay in antidepressant effects with traditional antidepressants, Zarate et al. (43) replicated the Berman et al. (42) study. Utilizing a double-blind study design, the authors determined that a single dose of 0.5 mg/kg ketamine administered to 18 patients with treatment resistant MDD produced a rapid and sustained antidepressant effect. Likely owing to the larger sample size and longer follow-up, other findings included that antidepressant effects were detected objectively at 110 min post infusion vs. 230 min in the Berman et al. study, and that antidepressant effects remained significant at 7 days post infusion vs. 3 days post infusion in the Berman et al. (42) study.

Price et al. (44) replicated the above studies with a larger sample size of 26 patents with TRD, with the addition of psychometric measures for the acute symptom of suicidality. The results of this study demonstrated that a single 0.5 mg/kg dose of ketamine hydrochloride solution administered over 40 min produced an improvement in depression symptoms at two h, and a significant response to ketamine at 24 h (71% met response criteria and 29% met remission criteria). The effect size of ketamine vs. placebo peaked at 24 h at 1.46, with a 95% confidence interval (CI, 0.91–2.01), with sustained effects after one week at smaller effect size (0.68; 95% (CI, 0.13–1.23). At two weeks, two patients still had antidepressant effects. These results are consistent with the findings of the above studies. Though subjects were not acutely suicidal, based on the data from this study, Price et al. (44) proposed that ketamine might be a safe effective emergency treatment for acute suicidality in a hospital setting. However, they noted ketamine's short-term transient effect.

In an effort to increase the duration of ketamine antidepressant responsivity and remission, aan het Rot et al. (45) enrolled 10 participants with TRD in a study of repeated-dose IV ketamine of six infusions over 12 days. The results of this supplemental study demonstrated that the repeated dose protocol was well-tolerated overall. Subjects reported that the psychotomimetic side effect symptoms remained of short duration and were not concerning. The average duration of antidepressant responsivity was 19 days after the last infusion, with a range of 6 to 45 days. One patient had only minimal depressive symptoms for 3 months after the course of treatment. Subsequent research replicated these results of improved duration of antidepressant effects with repeated ketamine doses (46–48). The results of these studies also revealed that adding a maintenance course of ketamine infusion treatment once per week to the repeated dose ketamine protocol had a cumulative and sustained antidepressant effect on TRD patients (49).

DiazGranados et al. (24) added to evidence for the efficacy of ketamine treatment by substantiating the hypothesis that ketamine has a rapid effect on the symptoms of acute suicidality. In their study, 33 TRD subjects were administered a single dose of 0.05 mg/kg IV dose of ketamine over 40 min. Results indicated a significant reduction of acute suicidality within 4 h of infusion. Additional studies examined the efficacy of ketamine on unipolar vs. bipolar depression. Results from these studies indicated that ketamine is also effective with bipolar disorder (16, 43).

These and other preliminary studies accelerated ketamine research that featured larger samples, improved research designs, more sophisticated outcome measures and new physiological and biological markers and measures that advanced understanding of the antidepressant mechanisms in ketamine as well as the contributory role of glutamate in mental disorders (35, 43, 46–54). Marcantoni et al. (55) conducted a comprehensive meta-analysis of intravenous ketamine treatment for treatment resistant depression studies. Twenty-eight studies from 2009–2019 across 35 journals were included in the analysis. Results indicated that ketamine had an effect on depressive symptoms within 4 h following a single infusion, with peak efficacy at 24 h. Ketamine efficacy was still present 7 days post infusion, and multiple infusions enhanced and prolonged ketamine efficacy.

Following Berman et al.'s (42) advice, considerable effort and resources have been expended in research focused on the investigation of and/or attempted synthesis of novel NMDA receptor antagonist substances with the rapid antidepressant qualities of ketamine but without the dissociative/hallucinogenic psychotomimetic side effects. This research has produced mixed results, with candidate drugs either producing dissociative side effects similar to ketamine, showing less rapid onset or only transitory effects, or exhibiting a narrower range of symptom efficacy (56). Efforts to develop ketamine substitutes based on other NMDAR antagonists such as MK-801, CGP 37849, and eliprodil appear to show anti-depressant effects in animal studies (57). Another line of research is developing drugs that mimic one of ketamine's mechanisms by targeting HCN channels or the I_*h*_ current passing through them, resulting in drugs such as Ivabradine/Procoralan, Lamotrigine/Lamictal, Gabapentin, and Guanfacine (36).

Of these, the most promising research direction is the still somewhat preliminary investigation of (R)-ketamine, an enantiomer of ketamine reportedly without psychotomimetic effects (58, 59). (R)-ketamine has produced antidepressant effects similar to those of racemic (R,S) ketamine and (S)-ketamine in rodent induced stress models of depression symptoms (57, 60, 61). Based on their study, Fukumoto et al. (61) suggested that the -R enantiomer may be responsible for ketamine's sustained antidepressant effects in treatment resistant subjects. Contradicting this conclusion is previous ketamine research results wherein (S)-ketamine enantiomer subjects (rodents) maintained antidepressant effects 32 days post induction even in the CUS-induced stress model, and the anhedonia rodent model (62).

Favoring (R)-ketamine is evidence of greater anti-depressant efficacy than either (R,S)-ketamine or (S)-ketamine in a study of nasal administration in humans (63). This is counterbalanced by evidence that (S)-norketamine, the metabolite of (S)-ketamine, was found to have greater antidepressant efficacy in rodents than its complement, (R)-norketamine, when tested in inflammation-induced and chronic social defeat stress models (64). Another argument for (R)-ketamine is evidence of lower levels of abuse risk in rodent models based on tests of self-administration, conditioned place preference, induced locomotor activity, induced psychomotor sensitization, and impact on metabolic activity and dopamine tone in the mPFC, as compared with (R,S)-ketamine or (S)-ketamine (65). Despite these results from rodent research, and its use as a recreational substance, ketamine is not considered a highly addictive drug (11, 12, 15, 17).

While ketamine's antidepressant efficacy has been well-demonstrated, there is as yet no clear evidence that either S+ or -R ketamine enantiomers are more effective or beneficial than racemic ketamine, or as discussed below, that separating ketamine from its dissociative effects is optimal or of any greater therapeutic value for TRD patients. Given ketamine's wide range of symptom relief within an even wider range of both medical and psychiatric disorders, focusing on the discovery of all mechanisms of action and their therapeutic interactions should remain the priority. The narrowly effective focus on a monoaminergic approach to the exclusion of complementary mechanisms of action leads to partial treatment response or no response with too many patients.

### Neuroplastogenic Effects of Ketamine

Current research agrees that dysfunctional glutamatergic neurotransmission is a major contributor in the underlying pathophysiology of depression (35, 41, 66–69), yet ketamine's antidepressant mechanisms of action are more complex than a blockade of NMDA receptors. The Fukumoto et al. (61) study contained supportive evidence that AMPA receptor stimulation is involved in the sustained antidepressant effects of ketamine (R, S) and ketamine enantiomer -R. Moreover, these researchers established that ketamine induced neuroplasticity in the PFC and hippocampus through modulation of the AMPA receptors, and further proposed that this activity may be ketamine's primary antidepressant mechanism of action (60, 61, 64, 70–73).

#### Ketamine, AMPA Stimulation, and Synaptic Regulation

AMPA receptor activities are involved in the regulation of synaptic function. Ketamine increases stimulation of the glutamate AMPA receptor, activating L-type voltage dependent calcium channels (VDCCs) and the activity-dependent release of BDNF, which in turn increases BDNF production in the PFC and hippocampus. BDNF is a critical regulator of mTOR signaling and synaptic plasticity, subsequently stimulating and increasing dendritic protein synthesis, spine maturation, and synaptic transmission (28, 74–78). Activity-dependent release of BDNF and the subsequent activation of the high affinity BDNF receptor TrkB (BDNF-TrkB) stimulates mTOR signaling via the activation of Akt and ERK pathways. The stimulation of mTOR signaling increases synaptic protein synthesis by repression of inhibitory binding proteins (4E-BP) and phosphorylating p70S6K (28, 30, 75, 76, 79). Ketamine induction suppresses NMDA receptor activities, which inhibits downstream eukaryotic elongation factor-2 kinase (eEF2K) and subsequent dephosphorylation of eEF2 (blocked the inhibition of BDNF translation), thereby augmenting BDNF synthesis (75, 80).

Increasing BDNF in the PFC and hippocampus is a vital component of the antidepressant effectiveness of ketamine. This theory was tested by introducing the Met allele (a single nucleotide polymorphism VAL66MET SNP knock in), which is a genetic BDNF mutation that blocks the activity dependent release of BDNF and effects 20–30% of humans (75, 80). VAL66MET is associated with several functional cognitive deficits in executive function and episodic memory. It is also associated with decreased hippocampal volume in both normal and depressed patients. Furthermore, it has been found to increase the predisposition to depression in patients previously experiencing trauma or the stress associated with childhood developmental trauma. Thus, VAL66MET SNP may be a genetic marker for susceptibility to depression, particularly for those who have suffered complex childhood trauma (75, 80).

VAL66MET SNP is only one of several BDNF-blocking heterozygous or partial deletion mutations (30). A negative regulator of the BDNF/ERK cascade, mitogen activated protein (MAP) kinase phosphatase-1 (MKP-1), is a dual specificity phosphatase that dephosphorylates both threonine and tyrosine residues whilst also a key regulator of the ERK signaling cascade (74, 81). In both rodent studies and human postmortem tissue studies, higher levels of MKP-1 in the hippocampus and related circuitry were discovered in depressed individuals vs. matched controls and in rodents exposed to CUS. This research is supportive of previous reports that MKP-1 is an immediate early gene that is activated by cellular stress and adrenal glucocorticoids (CUS and/or developmental trauma) and that predisposes to depression (74).

Li et al. (28) examined the cellular signaling pathways that mediate the behavioral actions of ketamine and focused on the signaling cascades known to rapidly influence synaptic plasticity. The authors hypothesized the rapid activation of mTOR signaling pathways may be the mechanism for the rapid acting antidepressant effects of ketamine. Ketamine's combined actions of synthesis and activity-dependent release of BDNF are contributing factors to ketamine effectiveness. Other mechanisms of antidepressant effect include ketamine's inhibition of GSK-3, another important brain protein linked to depression, by increased phosphorylation. Ketamine's antidepressant effects may also involve modulation through cortical GABA levels (75, 82). Furthermore, there is evidence that ketamine reduced depression-like behaviors in rodents following the induction of unpredictable stress, most likely by increasing γ-aminobutyric acid (GABA) levels in the anterior cingulate (74, 75, 83).

At this point, the focus shifts from effects on brain chemistry to ketamine's effects on brain structure and function through rapid induction of neuroplasticity via synaptogenesis and normalization of brain connectivity—topics that have already surfaced in the preceding discussion.

#### Ketamine and Synaptogenesis

The formation of dendrites and dendritic spines synapses, also referred to as synaptogenesis, is the basis of brain growth and brain connectivity. It is vital for the regulation and communication of information within neural networks, including the processing and incorporation of new information from either external sensory or internal sensory neural and/or chemical sources, and is essential for the prediction and adaptation of future responses. This process is an elementary component of neuroplasticity (30, 84). Research supports the proposition that functional and structural changes at spines and synapses are the basis of learning, memory, and cognitive flexibility (30, 85, 86).

TRD has a profoundly damaging impact on brain growth and connectivity, particularly in the prefrontal cortex (PFC) and the hippocampus. Sophisticated neuroimaging of the depressed brain revealed an intrinsic brain disconnectedness and significant loss of volume in the limbic region of the brain, in particular the PFC and the hippocampus when compared to a neurotypical brain (85–87). The limbic region of the brain is associated with the regulation of mood, emotion, and long-term potentiation associated with learning, memory, and cognitive flexibility (30, 67, 85, 88). Research supports the hypothesis that reduced volume in the depressed brain is related to atrophy of neurons in the limbic region of the brain including the PFC and the hippocampus.

Decreased hippocampal synaptogenesis is an alteration in the complexity of the architecture of the dendritic arbor of neurons, including the loss of dendrites and dendritic spines, a decline in the density and length of dendritic spines, and the number and length of surviving dendrite branches (30, 88, 89). Furthermore, postmortem reports of brain structure in depressed individuals revealed a reduction in size of neurons and glia and decreased levels of synaptic proteins (30, 90, 91). The discovery of ketamine and other substances that are capable of stimulating neuronal growth, particularly in areas of the brain depleted by pathology, is extraordinary and may substantially impact how mental illness and other brain pathologies are understood and treated.

The therapeutic effects of ketamine on complex neurocircuitry networks impaired by TRD occur on both micro and macro levels. Some mechanisms of both inter/intra-actions are not yet fully understood, but current peer reviewed literature has established that ketamine causes a rapid induction of synaptogenesis and neuroplasticity in the PFC (28–30, 74, 76, 85, 92, 93).

For example, Li et al. (28) reported that the administration of 10 mg/kg infusion of ketamine induced a rapid increase in the number of dendritic spines in layer V pyramidal neurons in the PFC of rodents at 24 h post ketamine induction. Furthermore, there was a simultaneous increase in the number of mushroom (mature) spines supporting the hypothesis that ketamine increased spine strength, stability, and function. It is important to note that activation of mTOR signaling was very rapid, occurring at 30 min post ketamine induction. However, while mTOR signaling was activated at low doses of 5–10 mg/kg, it was not activated at anesthetic doses of 80 mg/kg. Analyzing the neurotransmitter-induced excitatory postsynaptic currents (EPSCs) in these neurons confirmed that ketamine increased the frequency and amplitude of both 5 HT and hypocretin-induced EPSCs, associated with cortico-cortical and thalamus-cortical connectivity. The study also showed that rapid induction of synaptogenesis was mirrored by behavior changes in the depression-symptom behavior rodent models, as measured by a significant decrease of immobility in the forced swim test (FST), decreased escape failure and latency to escape in the learned helplessness paradigm, and decreased latency to feed in the novelty suppressed feeding test (NSFT). These antidepressant effects were sustained for 7 days.

The relationship between depression and stress, with its accompanying brain changes, has been well-established over the past 20 years in both preclinic and clinical trials (84). Excessive stress from either the external environment (exteroceptive) or internal environment (interoceptive) on the brain are known precursors of mood disorders, including depression. The type of stressor, the intensity and duration of the stressor, and at what brain developmental stage the stressors occur are all factors in depression pathology (84). Accordingly, research with novel anti-depressive pharmacology for depression typically begins with animal stress models, as this is the model that most closely simulates the depressive paradigm.

In TRD research, the gold standard is the chronic unpredictable stress (CUS) conditions stress model, as it is the model that induces symptoms most analogous of the symptoms of TRD and is also the best reflection of human developmental stress/trauma (94, 95).

The exposure of rodents to different stressors is a common experimental model for inducing specific depression-type behaviors related to core depression symptoms. Studying the behavioral changes in animal models induced by antidepressant substances is a systematic, efficient, and standardized way of assessing the efficacy of different substances and a necessary precursor to human trials (28, 29, 74–76, 84, 92–97). In studies of this type, ketamine is acknowledged to rapidly reverse the atrophy of PFC neurons caused by complex stress and other factors intrinsic to TRD (30).

For example, in a study that illustrates the association between childhood or developmental trauma, TRD, and the efficacy of ketamine, Li et al. (76) exposed rodents to the CUS condition for 3 weeks, a behavioral model reflective of the stress from childhood developmental trauma. In response, the animals exhibited decreased density of spines in layer V pyramidal neurons, decreased 5HT- and hypocretin-induced ionic, excitatory postsynaptic currents (EPSCs) in the same neurons. In addition, the rodents also developed anhedonia from CUS exposure—a core symptom of depression often associated with TRD. In this study, anhedonia was measured by a decreased preference for sucrose sweetened solution in the sucrose preference test. Additionally, the rodents developed an increase in the latency to feed in the NSFT test (anxiety model). Li et al. (76) administered a single dose of 10 mg/kg infusion of ketamine, which rapidly (within hours) reversed all CUS effects including structural, functional, and behavioral impacts. These effects were sustained for 7 days.

In a related rodent chronic stress study, Moda-Sava et al. (29) reported evidence that ketamine selectively restored spines lost during chronic corticosterone stress (CORT), restoring the number and function of PFC and hippocampal spines by means of selectively rescuing only the dendritic spines clustered on specific dendrite branches eliminated by chronic CORT treated mice. The authors found that 47.7% of newly formed spines were located < 2 μm from a spine lost during chronic CORT exposure, thus restoring the number and function of the affected prefrontal cortex and hippocampal dendritic spines. This action restored coordinated activity and rescued disrupted microcircuitry. Notably, ketamine did not increase the number and functions of spines randomly, but selectively restored only the spines lost during the stress exposure paradigm (28, 29).

This spine remodeling, also referred to as spinogenesis, occurred after the positive behavior effects of ketamine were present, and therefore could not be involved in the initial rapid (within hours) behavioral antidepressant response (29). However, behavioral differences in rodents measured at 2 to 7 days after ketamine treatment by Moda-Sava et al. (29), and at 27 days post treatment by Li et al. (28), were correlated with spinogenesis activity in those same time ranges. Though spinogenesis is not involved in the rapid acting antidepressant effects of ketamine treatment, it is likely intrinsic to ketamine's profile of sustained behavioral and antidepressant effects after ketamine vacates the body. The spine remodeling is in turn dependent on the circuitry restoration associated with the rapid effects of ketamine (28, 29).

Elhussiny et al. (98) conducted a study supporting earlier research by Li et al. (28, 76) in the premise that the CUS mouse model of depression produced rat brain atrophy and maladaptive remodeling of dendrites in the same brain regions affected in humans with TRD. Elhussiny et al. (98) also indicated that ketamine induction selectively reversed the atrophy and maladaptive dendrite remodeling of CUS exposure. In this study, male rats were exposed to unpredictable stress for five weeks to induce stress symptoms associated with TRD. CUS exposure produced hypofunction of activity-dependent glutamatergic synaptic transmission chains of events, and mGlu2 underwent a stress-induced selective downregulation in stress vulnerable animals. Elhussiny et al. (98) demonstrated that ketamine dosed at 10 mg/kg in CUS vulnerable animals produced selective modulations of AMPA GluA2 expression at synaptic membranes that reversed the effects of CUS exposure. The researchers suggested that this evidence confirms the AMPA GluA2 functional role in regulating stress vulnerability and, for the first time, proposed an mGlu2 involvement in the fast antidepressant effect of ketamine.

This evidence is consistent with the rodent data findings in the studies of Li et al. (28, 76) and Moda-Sava et al. (29) demonstrating that depression symptom relief occurred as early as 2–4 h post ketamine treatment, and spinogenesis was not detected until long after the positive behavior effects of ketamine were present, peaking at the 24–48-h mark. Therefore, spinogenesis did not appear to be involved in the initial rapid behavioral antidepressant response. As noted, spinogenesis is closely associated with ketamine's sustained effect on depression-related behavior.

Contrasting with the above analysis of peak spine modulation at 24 h post ketamine induction, there is new evidence of ketamine-induced rapid spine formation (< 3 h) under specific circumstances, that may be related to sex differences in depression etiology and ketamine effects. Zhang et al. (99), in their study of adult male, C57BL/6 mice aged 8 weeks and adult male, CD1 (ICR) mice aged 13–15 weeks that were previously assessed as susceptible to chronic social defeat stress (CSDS), found that (R)-ketamine (at 10 mg/kg) rapidly (<3 h) ameliorated the decreased spine densities in the prelimbic cortex (PrL) regions of the mPFC, CA3, and DG of the hippocampus in CSDS-susceptible mice. Furthermore, (R, S)-ketamine (or [R]-ketamine; at 10 mg/kg) rapidly (<3–4 h) attenuated the increased immobility time of TST in CSDS-susceptible mice. The potential association of this rapid spine formation with changes in depression-related behavior remains to be assessed.

The previous study also points to the fact that a variety of ketamine's effects need to be considered in light of sex-related differences, particularly as there are sex differences in depression etiology (100). For example, Zhang et al. (101) determined that there are sex-related differences in the effects of ketamine on locomotor activity and neuroplasticity-related protein levels in the brain. Results demonstrated that IV ketamine produced dose-dependent and region-specific effects on male and female rats: 10 mg/kg significantly reduced locomotor activity both in male and female rats, indicating sedative effects without severe dissociation. At 40 mg/kg ketamine IV infusion only stimulated locomotor activity in female rats. Other sex-related differences were found in the in c-Fos and pERK levels in the PFC and amygdala as female rats were more sensitive to ketamine-induced c-Fos elevation in these regions as compared to male rats. Female rats were further differentiated by high estrogen (proestrus/estrus phases) or low estrogen (diestrus phase). High estrogen females exhibited greater elevation of c-Fos compared to the low estrogen levels. Taken together, the current findings suggest that IV ketamine produces sex-related differences in spontaneous locomotor activity and neuroplasticity-related proteins in multiple brain regions of male and female rats. This is consistent with previous sex differences related to rodent studies of ketamine efficacy in that females exhibited higher behavioral ketamine efficacy rates than males, at the same or lower dosages (102–104).

Sex-related differences were also found by Sakar et al. (105), who examined the effects of ketamine on anhedonia and depression-like behavior, spine density, and synaptic proteins in both male and female rats subjected to the chronic isolation stress rodent model of depression (IS). Secondly, they considered the effects of ketamine (0, 2.5 mg/kg, and 5 mg/kg) on behavior and levels of synaptic proteins synapsin-1, postsynaptic density protein 95, and glutamate receptor 1 in both male rats and female rats in diestrus and proestrus phases of their estrous cycle. They also investigated ketamine effects on mPFC spine density in all groups.

Results indicated that male rats showed anhedonia and depression-like behavior after 8 weeks of IS, concurrent with decreases in spine density and levels of synapsin-1, postsynaptic density protein 95, and glutamate receptor 1 in the medial prefrontal cortex (105). These changes were reversed by a single injection of ketamine (5 mg/kg). At 11 weeks IS exposure, female rats showed depression-like behavior but no signs of anhedonia. Both doses of ketamine rescued depression-like behavior in the female rats. However, the IS associated reduction in synaptic proteins and spines in diestrus female rats could not be reversed by either dosage of ketamine. Other results showed that spine density was higher in female rats during proestrus than in diestrus. The authors concluded that there are sex related differences in the etiology of depression as well as in the antidepressant effects of ketamine in synaptic proteins synapsin-1, postsynaptic density protein 95, and glutamate receptor 1 and mPFC spine density in male rats subjected to social isolation models in that male but not female rats responded to treatment. The authors suggest there may be different underlying mechanisms for efficacy of ketamine in the two sexes.

While the great majority of published evidence for ketamine-induced synaptogenesis and neuroplasticity comes from animal models, a recent study has successfully translated some of these important findings into studies with human subjects (94). Höflich et al. (94) conducted a double-blind study with 31 healthy subjects (14 F/17 M; mean age 25.2), measuring increases in overall hippocampal subfield volume in CA1 (*p* = 0.009) from baseline to 1 h after administration of a bolus of 0.11 mg/kg maintained by infusion of 0.12 mg/kg over 20 min. This study shows rapid pro-neuroplastic effects in the hippocampus, highlighting the key role of the hippocampus in ketamine's mechanisms of action. It will be important to further the translation of findings regarding ketamine's impact on synaptogenesis from animal models to research with human participants.

#### Normalized Brain Connectivity

Ketamine is also associated with normalized connectivity in the default mode network (DMN). Past research has demonstrated a clear intrinsic abnormal neural connectivity in the DMN in depressed individuals (85). The triple network model of dysfunction is a hypothesis in which the underlying etiology of depression is the disruption of connectivity within the DMN, the salience network (SAL), and the central executive network (CEN). In depressed individuals, the DMN, the network responsible for introspection and rumination, is significantly overactive, accompanied by significant underactivity in the SAL, the network responsible for processing salient material from the external world, and the CEN, which is responsible for working memory and attention.

One of the relevant impacts of ketamine's AMPA stimulation, the downstream enhancement of neurotropic factors such as BDNF and the resulting synaptogenesis, is a normalization of connectivity within and between multiple brain areas. For example, there is evidence that in certain conditions, with appropriate ketamine dosage, and at certain times post-treatment remitters showed temporary normalization (106) in global brain connectivity (GBC) between salience networks and the default mode network (DMN) (85); and between the dorsal caudate and the ventrolateral PFC (18); normalized connectivity between the amygdala and the subgenual anterior cingulate cortex (sgACC) (107); between the posterior cingulate and the right posterior insula; between left and right primary somatosensory cortex, between left frontal cortex and left primary somatosensory cortex (108), and between anterior insula to anterior cingulate (109); normalized global grain connectivity with global signal regression (GBCr) in the PFC (110), cerebellum, caudate, and insula (111); normalized connectivity in the sensorimotor and salience networks (112), in the parahippocampal gyrus, cingulate gyri, left medial and middle frontal gyri, and precuneus (113), in the right caudate (114), and in the sgACC (115).

GBCr is an innovative graph-based analysis that produces an assessment of whole-brain connectivity (functional connectivity strength) without the need for a priori seed selection areas, as measured by resting-state blood-oxygen-level-dependent (BOLD) fMRI, an *in vivo* imaging technique for mapping large-scale brain networks. Although there appears to be some difference of opinion, there is significant evidence that GBCr is a valid biological marker of brain networks. For example, several studies have found correlation between GBCr and normal brain functions including cognition as well as correlation with regional cerebral blood flow. GBCr has also been used to map major neuronal networks and help identify network abnormalities involved in neuropsychiatric disorders (116).

Abdallah et al. (111) conducted a study utilizing GBCr to quantify functional dysconnectivity measured via resting-state (BOLD) fMRI in 25 healthy control participants (HC) and 18 treatment-resistant MDD patients (TRD). All participants were examine using GBCr at baseline and 24 h. post a single dose 0.5 mg/kg intervenes infusion of ketamine over 40 min. TRD patients were in an active depressive episode during baseline pre ketamine measures. The TRD patients were then divided into responder and non-responder (values established on symptom reduction) groups for analysis. Overall results of this study showed MDD patients with widespread whole brain dysconnectivity with reduced GBCr primarily in the prefrontal cortex and increased GBCr in the posterior cingulate, precuneus, lingual gyrus, and cerebellum, as compared to HC participants at baseline. Post treatment results revealed that although HC participants returned to baseline, MDD patients had significant increases of GBCr in the right lateral PFC and reductions in the left cerebellum, indicating that ketamine treatment rapidly normalized areas of brain connectivity in MDD patients. These results were confirmed with similar data findings in a follow-up seed-based analysis. Other results from responder and non-responder group comparison *t*-tests showed no differences pre ketamine treatment. Post treatment comparisons revealed that responders had higher increases of GBCr in the prefrontal cortex, caudate, and insula than non-responders. Furthermore, ketamine responders had higher delta GBCr values in the right lateral PFC and the left anterior insula (111).

However, there has been some controversy regarding whether normalized connectivity is caused by a ketamine-induced glutamate surge, or whether normalization is a secondary result of antidepressant response. A study by Abdallah et al. (116) demonstrated the relationship between the glutmate surge and increased connectivity by showing that this surge begins at ketamine infusion and is temporally associated with normalized PFC GBCr; the authors posited that this acute glutamate surge may be a predictor of treatment response.

A subsequent study by Abdallah et al. (110) used GBCr to compare the efficacy of ketamine, lanicemine as an alternate NMDAR antagonist, and placebo in normalizing brain connectivity in 56 MDD patients. Measures were taken at baseline, during infusion, and at 24 h post-treatment. Results indicated that ketamine increased PFC GBCr during infusion and remained present at 24 h post-infusion. Neither lanicemine nor placebo produced an effect on connectivity during administration or at 24 h post-administration, further supporting glutamate surge as the mechanism associated with ketamine as a rapid-acting antidepressant.

In contrast with the above findings, Kraus et al. (117) was unable to replicate previous findings of reduced or enhanced connectivity in MDD individuals as compared with healthy controls, in multiple brain areas, as found by Abdallah et al. (111). Reduced deviations from normal connectivity were found in similar brain regions in MDD participants, but in the case of the midcingulate cortex the direction of difference was opposite. Kraus et al. (117) also found no differences in GBCr post ketamine administration, as compared with baseline and placebo. However, given that Kraus et al. (117) performed fMRI scans 2 days post-treatment rather than at 24 h as with Abdallah et al. (111), their result likely shows the short duration of some connectivity changes in response to ketamine. In addition to differences in experimental timing, Kraus et al. (117) noted that divergences in findings may also be affected by differences between their study and Abdallah et al. (111) in participant age, gender balance, body mass index, time from initial diagnosis, statistical standards, preprocessing methods and software, and experimental conditions. In any case, studies such as Kraus et al. (117) temper expectations of early consensus.

Normalized brain connectivity is associated with enhanced neural plasticity, which is also a demonstrated effect of ketamine treatment.

#### Neural Plasticity

Synaptogenesis and brain connectivity are both elements of neural plasticity, the ability to change and adapt in response to stimuli that is crucial to normal, healthy brain function (118); neural plasticity is associated with critical periods of postnatal life, with plasticity gradually decreasing as development progresses toward an adult brain. The common belief in the past was that the adult brain became developmentally static and incapable of neuroplasticity other than adaptation in response to stimuli. However, this proved to be somewhat untrue. As neuroscience research has progressed, it has become apparent that the adult brain retains some capacity for adaptation—though not at critical period levels. In addition, it is now clear that some substances are capable of rapidly inducing neural plasticity in mature adults. The implications of these findings are far reaching and consequential, as it means that there may be some potential for recovery among individuals who suffer from various brain deficits and neural psychiatric disorders including mood, anxiety, substance abuse, trauma, and personality disorders (118).

Olson (118) coined the term psychoplastogens to describe fast acting (24–48 h) compounds with the ability to precipitate structural and functional reorganization of neural circuits that facilitate positive and sustained behavioral change. Psychoplastogens are a new class of rapid-acting antidepressants such as ketamine and other more traditional psychedelics including LSD, DMT, MDMA, and psilocybin. Psychoplastogens reactivate a state of plasticity in the adult neural cortex that closely resembles the enhanced plasticity normally observed during postnatal critical periods. Induced neural plasticity, or I-plasticity, is a recent term used to describe induction to a state of juvenile-like plasticity, thereby improving the brain's capacity to respond to both internal and external stimuli and to improve or repair underlying developmental or physiological malfunctioning networks (119–124).

Electrophysiological studies have pointed to an increase in plasticity in response to ketamine treatment in human subjects, as evidenced by long-term potentiation (LTP) and increased gamma power [e.g., (109, 125)]. However, in a 2019 magnetoencephalography study, Nugent et al. (107) found that subjects with lower gamma power at baseline and higher gamma power after ketamine treatment were associated with better antidepressant response; conversely, in subjects with higher gamma power at baseline, the results were inverted—suggesting that this mechanism of action may show some variance between different biological subtypes.

Gálvez et al. (126) conducted the first human pilot study using fMRI to measure neuroplasticity-induced changes pre and post ketamine treatment, with three subjects diagnosed with treatment resistant depression. Neuroplasticity was measured by cortical activity in the motor cortex. Subjects received either 8 intranasal treatments of 100 mg ketamine or 4.5 mg midazolam. Mood ratings were performed by a trained blinded rater at baseline and 24–48 h after the ketamine course, using the MADRS. Neuroplasticity was assessed in the motor cortex using a PAS paradigm at baseline and 24–48 h after the administration of either ketamine or midazolam. Results indicated that the subjects receiving ketamine, but not those receiving midazolam, presented a marked increase in neural plasticity after the treatment course. However, mood changes were not associated with changes in neural plasticity. This is consistent with rodent model data findings that depression symptom relief occurred as early as 2–4 h post-ketamine treatment, and spinogenesis did not occur until long after the positive behavior effects of ketamine were present, peaking at the 24–48-h mark (28, 29, 76).

Recent research combines electroencephalography (EEG) technology with fMRI and phMRI in ketamine studies, providing both complementary information and aiding the interpretation of fMRI data (127). Both fMRI and resting-state fMRI (rsfMRI) are direct non-invasive ways to examine ketamine effects on the brain, acquiring full volumes of the brain at various time points during and after ketamine infusion. EEG has been instrumental to the investigation of real time neural activity, functional connectivity, and mapping the organization and interactions of neural networks (128). EEG is also sensitive to changes in LTP, which is a reliable correlate of neural plasticity (128).

Research with ketamine and other substances that induce neuroplasticity is growing rapidly within the diverse fields of mental health, traumatic brain injury (10, 129, 130), degenerative brain disorders such as Parkinson's and Alzheimer's (131, 132), and neurodevelopmental disorders including autism (133, 134) and schizophrenia (135). Some of the discussed impacts of ketamine may also be viewed through the lens of ketamine's anti-inflammatory effect on inflammation-induced symptoms of depression.

### Inflammation, Depression and Ketamine

Immunologic processes and inflammation may play a pivotal role in the development and maintenance of psychiatric disorders. Inflammation can disrupt monoaminergic and glutaminergic systems to create dysfunctional brain networks. That is, inflammation influences glutamate release, transmission, and metabolism, resulting in accumulated extracellular glutamate in the central nervous system. Inflammation has an inhibitory effect on monoamine metabolism and a resultant decreases BDNF. Thus, inflammation is associated with a poor response to conventional antidepressants, whose efficacy relies in part on increasing monoamine availability and inducing BDNF and synaptogenesis (136). Persistent low-level inflammation, from multiple sources including childhood stress, chronic stress, psychological distress, and chronic inflammatory diseases such as autoimmune disorders, is implicated in depression. While depression is not considered an inflammation disorder, some forms of depression—particularly treatment resistant forms—result from infections, gut bacteria, and other inflammation-causing medical disorders. In such cases ketamine appears to play an antidepressant role by regulating peripheral and central immune systems, including peripheral inflammatory cytokines, central microglia, and astrocytes (12).

The anti-inflammatory properties of ketamine and its downstream effects have sparked research interest. Chronic stress causes inflammation, and a portion of ketamine's antidepressant effect has been linked to its anti-inflammatory effect, in the form of a down-regulation of pro-inflammatory cytokines (12). However, this down-regulating cascade is not a mandatory outcome of ketamine treatment but is only triggered when chronic stress-induced inflammatory processes are present (137). To test whether ketamine's anti-inflammatory effect is one of its mechanisms of action Yang et al. (138) randomly divided 20 Winstar rats into two equal groups of either saline or ketamine induction. Ketamine effects and interleukin (IL)-1β and IL-6 were measured in the prefrontal cortex and hippocampus of a stress exposed rat-model. The ketamine group demonstrated a decrease in IL-1β and IL-6 in rat prefrontal cortex and hippocampus compared with the saline group (*p* < 0.05). Thus, ketamine-induced antidepressant effects are associated with decreased levels of IL-1β and IL-6 in rat prefrontal cortex and hippocampus, suggesting that ketamine's mechanism of action is associated with its impact on the inflammatory response in the central nervous system.

In a later study Wang et al. (139) equally randomized 48 rats into one control group and five groups of rats exposed to chronic unpredictable mild stress (CUMS). Either saline or 10 mg/kg ketamine were administered. The results showed that CUMS exposed rodents exhibited depression-like behaviors and up-regulated the hippocampal levels of IL-1β, IL-6, TNF-α, IDO, and the kynurenine/tryptophan ratio, which were then attenuated by a subanesthetic dose of ketamine. The authors concluded that the downregulation of pro-inflammatory cytokines is associated with ketamine's antidepressant effect in the rat hippocampus (139). However, in subsequent clinical experiments the relationship between ketamine and reduction of inflammatory factors were somewhat inconsistent, highlighting the need for more research in this area.

In addition to the effects previously discussed, ketamine can also impact the brain by inducing high-entropy brain states that may provide opportunities for adaptive reorganization.

### Ketamine-Induced Entropic Brain States: An Opportunity for Adaptive Reorganization

Depression is not just about chemical and neurological changes, it is also about the way experience, beliefs, and self-concept are organized (140, 141). Tart (142) has proposed that states of consciousness constitute distinct patterns of overall mental functioning associated with “interlocking sets of rules and theories” (p. 1204) by which experience is organized. Carhart-Harris et al. (31) have claimed that psychedelics allow the deliberate neurobiological manipulation of states of consciousness in subjects, potentially disrupting habitual mental organization in a beneficial way; when coupled with recent advances in neuroimaging, electroencephalogram (EEG), and transcranial magnetic stimulation as real-time physiological and biological measures of entropy, the psychedelic experience generated by serotonergic psychedelics as well as ketamine is shown to be related to entropic brain states (143, 144).

The definition of entropy in this context is the degree of randomness or disorder in the brain at any given point. Applying the principles of physics, biology, and psychology, the entropic brain theory is a neuroscientific interpretation and explanation of the mind, including conscious and unconscious states. Low entropy, which the authors identified as secondary consciousness, is associated with higher organization, or less randomness, and is correlated with adult, mature, and awake states of consciousness; a high state of entropy, which the authors identified as primary consciousness, is associated with less organization, or more randomness, and is correlated with more primitive, pre-adult, unconscious, and/or sleeping states of consciousness.

While this new interpretation of data requires additional study, Carhart-Harris et al. (31), through a rigorous body of complex scaffolded research, have documented that the introduction of psychedelics alters brain function via the disruption of the connectivity between the medial temporal lobe (MTL), which is comprised of the olfactory cortex, the amygdala, and the hippocampus, and the cortical regions of the DMN, including the mPFC, posterior cingulate cortex, inferior parietal lobule, lateral temporal cortex, hippocampal formation, and the precuneus. The connection of the MTL and the DMN is required for the maintenance of secondary consciousness (low entropy), healthy awake and adult behavior paramount to metacognition. Therefore, because psychedelics disrupt the hippocampal-DMN network, a regression to the primary consciousness (high entropy) occurs. The effects are transient with a quick refractory period and are potentially conducive to the reorganization and adaptive rewiring of neural circuitry that may have been damaged in the TRD patient.

This concept is likely relevant to the effect of psychedelic psychotherapy on depression. Depression is notably characterized by an atypical rigidity of usually highly self-critical negative thoughts, behaviors, and beliefs manifesting in feelings of hopelessness, helplessness, anhedonia, and isolation (145), Within the context of brain entropy theory, depression is possibly an overabundance of secondary consciousness or an abnormally low entropy state. The introduction of psychedelic therapy introduces a regression to a primary, high entropy brain state and associated state of consciousness, disrupting the depressive stereotypical thoughts and behaviors by disintegrating their very foundation. For a more thorough comprehensive explanation of a complex theoretical construct, please refer to Carhart-Harris et al. (31) and Carhart-Harris (143).

The importance of this potential mechanism of action is not only its possible efficacy, but also that it may point to a need to extend the current discrete nosological psychiatric model, in which disorders are solely attributable to brain dysfunction, toward an understanding of mental disorders as multi-dimensional processes that include mental injury from adverse environmental events and trauma, and that may benefit from the therapeutic potential of altered states (146). Given that more individuals are affected by mental illness than ever before, psychedelic assisted psychotherapies that engage entropic brain states show promise as a rapid, safe, and broadly effective treatment modality, even with treatment resistant mental disorders.

Although there is preliminary evidence that the psychedelic ketamine experience is associated with increased entropy in the form of increased spontaneous magnetoencephalographic (MEG) signal diversity (144) and subanesthetic doses of ketamine have produced elevated complexity as measured by EEG (147), conclusions regarding this potential mechanism of action by ketamine relies in large measure on research done on entropic states induced by psilocybin or LSD—which is itself at a preliminary stage. As such, it is not yet clear that the initial results obtained with these other psychedelics can be generalized to ketamine.

### Potential Therapeutic Impacts of Ketamine's Psychedelic Phenomenology

There is a body of research which supports the premise that the quality or intensity of a psychedelic experience is associated with better therapeutic outcomes (11), without respect to the specific psychedelic substance. Some of this evidence is confounded by the different treatment methods associated with psychedelic assisted psychotherapy. Pahnke (148) and Grof (149), two major contributors to the contemporary theoretical constructs of the phenomenological psychedelic experience, both agree that the psychedelic experience may (but not always) include a highly intense episode—or, as Pahnke (148) termed it, *a psychedelic peak experience* (p. 176). Furthermore, experiencing this psychedelic peak event improved therapeutic outcomes in psychedelic assisted psychotherapy. Patients who have experienced a psychedelic peak experience have rated it as one of the most meaningful events of their life (32, 148–155).

Pahnke (148) has proposed that nine characteristics are involved in a psychedelic peak experience: a sense of unity; the transcendence of time and space; a deeply felt positive mood; a sense of sacredness; the noetic quality; and paradoxically alleged ineffability, transiency, and persisting positive changes in both behaviors and attitudes toward self, others, life, and the experience itself. Additionally, research suggested a feel-good factor effect occurred wherein subjects experienced an afterglow effect that lasted for approximately 2 weeks to 1 month (32). Pahnke (148) characterized the afterglow effect as an elevated and energetic mood with relative freedom from concerns from the past and from both guilt and anxiety. It is interesting to note that the duration of the afterglow effect of 2 weeks to 1 month is consistent with the duration of the antidepressant effects of ketamine on TRD subjects post treatment, long after the ketamine has vacated the body.

Supportive evidence for the correlation between peak psychedelic/mystical experiences and improved therapeutic outcomes comes from a series of two sets of double-blind studies with high doses of psilocybin administered in a therapeutic supportive environment (150–152). Results showed evidence that having a psilocybin-induced mystical experience effected a profound positive personality change in 32 and 17 psychedelic naïve adult subjects, respectively. Longitudinal data from the same studies confirmed that those positive personality changes persisted over 14 months.

In a later study with ketamine, Sos et al. (34) conducted a double-blind, placebo-controlled crossover study to examine the relationship between psychotomimetic symptoms and antidepressant efficacy. In the study, 27 hospitalized TRD patients were administered a single 0.5 mg/kg dose of ketamine and monitored over a two-week period. The results confirmed that a higher intensity of psychotomimetic symptoms experienced during ketamine infusion correlated with a higher alleviation in mood ratings throughout the following 2 weeks, with peak efficacy at day 7. Furthermore, researchers concluded that ketamine's substantial efficacy with TRD mediated through its effect on NMDA receptors may be exponentially enhanced by its psychotomimetic effects.

Dakwar et al. (19) conducted a double-blind randomized inpatient study on the efficacy of ketamine on the reduction of cocaine dependency of eight cocaine-dependent subjects with a history of cue-induced cravings who were not actively seeking treatment. Since ketamine engenders transient alterations in consciousness, the researchers also evaluated whether the altered state of consciousness influenced the efficacy of the treatment, which was measured by standardized psychometric tests of motivation to quit cocaine and a reduction of cue-induced cravings. All subjects received three counterbalanced infusions, each 48 h apart. One infusion of 2 mg of lorazepam as active placebo, one infusion of 0.41 mg/kg of ketamine, and one infusion of 0.71 mg/kg of ketamine. Within 15 min of infusion subjects completed measures of dissociation, the Clinically Administered Dissociative Symptoms Scale (CADSS), and a mystical effects scale adapted from Hoods Mysticism Scale (HMS) at baseline and at 24 h post infusion.

Results indicated that the experience of mystical type phenomena with ketamine infusion is dose dependent, with no mystical phenomena generated with lorazepam, and little effect at 0.41 mg/kg ketamine infusion (19). Additionally, the intensity of the transient mystical phenomena resolved completely at 24 h post infusion and the therapeutic effects of ketamine on the motivation to quit cocaine, but not on cue-induced cravings. These data confirmed the understanding from previous research that (a) experiencing a psychedelic peak experience is dose dependent, and (b) experiencing a peak psychedelic experience may be a key mediator of change in mental health, including addictions.

These studies provide evidence of a relationship between peak psychedelic/mystical experiences and improved therapeutic outcomes. Additional evidence was offered by Roseman et al. (155) in a study on psilocybin efficacy for TRD, in which researchers concluded that “the quality of the acute psychedelic experience is a key mediator of long-term change in mental health” (p. 974). In this study, 20 subjects diagnosed with TRD underwent 2 psilocybin treatments separated by 1 week. The first treatment consisted of an oral dose of 10 mg and the second treatment a dose of 25 mg. All participants completed the altered states of consciousness questionnaire (ASC). Within the ASC, the incidence and intensity of the dimensions of oceanic boundlessness (OBN), which correlated to mystical experiences, and the dimension of dread of ego state dissolution (DED), which correlated to anxiety, predicted positive therapeutic outcomes, whereas the dimensions that measured sensory/perceptual effects did not (p. 977). The degree of TRD symptoms was rated using the Self-Report Quick Inventory of Depressive Symptoms (SRQIDS). Measures were taken at 1 day, 1 week, and 5 weeks. Results indicated an overall positive therapeutic outcome and positive therapeutic outcomes across all time periods with a significance of *p* = 0.001 to *p* = 0.002, respectively. Secondly, there was a positive correlation between the occurrence and intensity of measures of OBN and DED and the degree of positive therapeutic outcomes.

Contrary to the previously described studies, not experiencing a mystical/spiritual event does not necessarily alter the overall positive effects of LSD. Liechti et al. (153) conducted a two-trial, placebo controlled, and double-blind crossover design LSD study using 100 mg and 200 mg doses with 24 and 16 healthy subjects, respectively. The first objective of the study was to measure the mystical experiences generated and the relationship to altered states, whereas the second purpose was to assess dose and blood concentration related to states of consciousness effects. The psychometric tools for measurement were the Mystical Experiences Questionnaire (MEQ) and the Altered States of Consciousness 5 Dimensions Edition (5D-ASC). Results indicated that LSD produced robust overall effects: the higher dose of LSD rated as high on the positive mood domains on the MEQ with a correlative high rating on the 5D-ASC blissful dimension. Although few participants reported having full mystical experiences on LSD, those with a dose of 200 mg scored substantially higher on bliss, insightfulness, and changes in meaning in perception and greater empathogenic effects than those subjects with 100 mg. The authors concluded that in contrast to previous studies, mystical experiences were not universally the most prominent feature of LSD experience. The authors claimed that these results call into question the definition of peak psychedelic experiences as an experience of a mystical or spiritual nature, at least in LSD experience.

Of more direct relevance, a study with 82 participants treated with ketamine for TRD did not find any association between the dissociative experience of “floating” while under the influence of ketamine, and ketamine's anti-depressant efficacy (156). However, given that there is a wide range of ketamine's psychotomimetic effects, lack of association with one particular type of experience does not negate a possible connection between intensity of experience and ketamine's anti-depressant effect [e.g., (34)]. By contrast, a study of 32 subjects with MDD who received either a single 0.44 mg/kg ketamine dose or the active placebo remifentanil (157) found that dimensions of altered states of consciousness associated with spirituality, experience of unity, and insight correlated with greater antidepressant response.

There is also evidence that the dissociative effects of ketamine, without reference to peak or mystical dimensions, correlate with its antidepressant effect, sometimes in a dose-dependent manner (158). Dissociative effects during administration may be markers of ketamine's therapeutic impact on network connections associated with rumination (159), self-monitoring and autobiographical memory (160–162), and emotionally valenced processing (46, 85, 163)—often areas related to key symptoms of depression such as excess rumination and self-focus, and negative bias in emotional processing. As such, efforts to follow Berman et al.'s (42) counsel to evade ketamine's dissociative effects deserves serious reconsideration as a possibly outdated goal.

Even with this ongoing question of the relationship between mystical and spiritual experience, ASCs, and psychedelic assisted psychotherapy, it is at least possible that ketamine, when prescribed at subanesthetic doses, offers a unique experiential therapeutic opportunity for TRD subjects. Becker (164) theorized that ketamine's psychological benefit and sustained effects may be exponentially enhanced due to its induction of a brief transformational state of consciousness and the consequent opportunity for psychotherapy to be more efficient. Wolfson (23) concurred and stressed that “ketamine assisted psychotherapy's value is inextricably linked with the psychedelic experience that ketamine induces” (p. 37). Although there is evidence in support of this sentiment, the empirical research specific to the efficacy of the psychedelic qualities of ketamine on TRD is lacking compared to other ketamine/depression peer reviewed studies.

Although ketamine (NMDA antagonist) and classic psychedelics (5-HT2AR agonists) have very different underlying mechanisms of action, they also have similarities that are neurobiological, cognitive, and psychological (165). Both substances appear to induce structural and functional neural plasticity through the same BDNF, Trkb, and mTOR signaling pathways. Ketamine and classic psychedelics similarly stimulate neural plasticity via BNDF overexpression due to mTOR activation. This activation results in high level network connectivity changes particularly within the DMN, which is associated with executive function and may allow for the possibility of long-lasting cognitive and psychological flexibility, which is in some senses the very definition of psychological health (165). Ketamine and classic psychedelics also induce similar altered states of consciousness and phenomenological experiences.

Despite these similarities and the possibility that ketamine and serotonergic psychedelics may share similar downstream mechanisms of action (166), caution should be exercised in generalizing from other psychedelic substances to ketamine until additional research can confirm that such findings also apply to ketamine.

### Risks and Side Effects

As ketamine becomes more widely utilized as a treatment intervention for depression, more data are needed on its acute and long-term safety in clinical settings, and the prevalence and impact of its side effects on therapeutic interventions. The most prominent risk for a state-altering substance such as ketamine is its abuse liability—the drug's potential for misuse outside of the therapeutic environment due to its psychoactive effect. Racemic ketamine has some demonstrated abuse liability in both animal and human studies, though there appear to be important differences between R and S enantiomers of ketamine: rats self-administered (S)-ketamine, but not (R)-ketamine, in subanesthetic doses; (S)-ketamine, but not (R)-ketamine, induced locomotor activity described as similar to opioid receptor-dependent behavior; (S)-ketamine also induced psychomotor sensation and conditioned place preference in mice, and selectively increased metabolic activity and dopamine tone in medial prefrontal cortex of rats (65).

However, there appears to be considerable disparity between the potential for risk abuse and the actual prevalence of abuse liability, since ketamine is not known or classified as a highly addictive drug (11, 12, 17). For example, of 163 participants screened for side effects in a retrospective review of NIH patients who received a single subanesthetic dose of ketamine, there were no post treatment drug cravings or need for recreational use (167). However, dependency and overuse can and does occur in unsupervised settings—often among polydrug users, and typically confined to parties or large music and dance events—which may result in serious long term medical complications (168). For example, chronic ketamine abuse can produce toxicity to the gastrointestinal and urinary tract including epigastric pain, hepatic dysfunction, impaired gallbladder activity, cystitis, and renal failure (169). Due to relatively low prevalence and dependency rates in most countries, accurate abuse levels for ketamine are difficult to determine. The situation may be different in China—allegedly one of the largest sources of illegal manufacture and export—which is reported to have one of the highest incidence rates of ketamine abuse; however, actual incidence rates of chronic ketamine abuse in China has not been reported (168).

With respect to unwanted side effects, a systematic review by Short et al. (170) aggregated and analyzed existing data on ketamine safety for use with depression in adult human clinical trials to date. A comprehensive literature search found 591 relevant studies, of which 60 were included: 20 randomized control trials, 17 open-label trials, 20 case series or reports, and three retrospective studies. The 60 studies analyzed included 899 patients. Acute and psychotomimetic, cognitive, physiological, and neurological side effects occurred most often immediately following intravenous ketamine administration and resolved within 90 min of treatment; all such effects dissipated within 4 h. Psychotomimetic side effects most commonly included dissociation, followed by perceptual disturbance, odd or abnormal sensation, derealization, hallucinations, feeling strange or unreal, and depersonalization. Cognitive side effects included poor memory/memory loss, poor concentration, confusion, and cognitive impairment/diminished mental capacity. Physiological effects included acute cardiovascular changes such as increased blood pressure and heart rate, palpitations or arrhythmia, chest pain/tightness/pressure, dizziness upon standing, or decreased blood pressure and/or heart rate. Neurological side effects included headache and dizziness (most common), followed by sedation or drowsiness, faintness/light-headedness, poor coordination/ unsteadiness, and tremor/involuntary movements. No long-term side effects were reported, though it is important to note that most studies were only conducted over a short period of time. Evidence supporting the absence of long term adverse ketamine effects comes from a retrospective study of associated side effects in National Institutes of Health (NIH) patients with treatment resistant depression who received a single subanesthetic ketamine infusion.

Acevedo-Diaz et al. (167) harvested data from four placebo-controlled, crossover ketamine trials and one open-label study over 13 years at the NIH clinical center involving 188 participants. Of those participants 163 were patients with major depressive disorder or bipolar disorder, and the other 25 were healthy controls. Screening was completed for 120 side effects through standardized measures and clinician evaluations, with 33 side effects found to be related to ketamine treatment; “feeling weird/strange or loopy” was the side effect most commonly noted by participants. One-half of patients who received ketamine experienced eight side effects: feeling strange, weird, or bizarre; feeling spacey; feeling woozy/loopy; dissociation; floating; visual distortions; difficulty speaking; and numbness. All side effects resolved within four h, and there were no reported or observed adverse effects, and no increase in ketamine cravings or abuse after administration. In the three month follow up data there were no reported or observed treatment related serious adverse events, no drug cravings, no propensity or disposition for recreational use, and no significant memory or cognitive impairments or long-lasting delirium.

## Discussion

Advances in neuroscience have transformed the perception of treatment resistant depression (TRD) from a monoaminergic neurotransmitter systems model in which there is dysfunction of a few specific parts of the brain, toward the discovery that depression is a complex network disorder (30, 84). Current research supported the hypothesis that depression etiology is likely the symbiosis of (1) genetic precursors; (2) environmental and biological stressors; (3) modulation of the glutamatergic system; (4) disrupted signaling pathways: (a) mammalian target of rapamycin, (mTOR), (b) tropomyosin receptor kinase B (TrkB), and (c) 5-hydroxy-tryptamine (5-HT2A) in the prefrontal cortex (PFC); (5) reduced expression of brain derived neurotrophic factor (BDNF); (6) altered structural and functional brain connectivity in neurocircuitry, especially in the default mode network (DMN); (7) inhibited neurogenesis; and (8) inhibited neuroplasticity (28, 29, 74–76, 85, 92, 93). All of these factors are interconnected and interrelated, both structurally and functionally. An alteration in any component causes a domino effect and results in abnormalities throughout the network (84).

The prospect of rapid-acting treatments with antidepressant effects at the receptor level, induced, targeted synaptogenesis at the neuron level, induced neural plasticity with improved connectivity and balance at the network level, high entropy brain states, and transcendent experiences at the subjective level, is highly promising. Ketamine's demonstrated efficacy with treatment resistant depression patients likely relies on the sum and synergy of these effects and mechanisms of action which, taken together, can inform a more integrative approach to ketamine therapy. Ketamine provides a complex set of mechanisms that respond effectively to the complex network of dysfunctions associated with TRD.

There are resulting opportunities to optimize ketamine therapy protocols in several ways. Not all of these suggestions may be feasible or desirable for any given treatment application, but together they may point toward ways ketamine therapy can be enhanced through consideration of each of the multiple levels of ketamine's mechanisms of action. For example:

1. Psychoeducational preparation of patients will be more effective when informed by an understanding of ketamine's multiple levels of beneficial effect—its receptor-based antidepressant effect; its neurogenesis-based support for lasting changes in brain, behavior, and experience; its impact on disordered patterns of brain network organization associated with treatment resistant depression, its stimulation of high entropy brain states that may enable neural and cognitive resets; and its induction of a dissociated state that may provide relief from negative affect and thought patterns and access to positive or even inspiring affective states.
2. Calibration of ketamine dosage to ensure induction of a high entropy brain state may provide an enhanced opportunity for adaptive neural rewiring, cognitive reorganization, and possible access to a peak, mystical, or otherwise meaningful experience. In Morin's (171) estimation, it is possible to determine optimal ketamine dosage by tracking the onset, maintenance, and duration of a high entropy brain state during each ketamine session utilizing real time EEG measurements. For example, if at the start of the session the patient is in a comfortable position with an eye mask, the dominant posterior alpha rhythm over the occipital cortex is present at high power. At onset of a dissociative state, the optimal dose can be titrated so alpha oscillations decrease in power and nearly disappear over the occipital cortex, indicating that an effective dose has been delivered. In this way, dosage can be individualized based on the level at which the individual enters and maintains a high entropy brain state, rather than based on mg/kg. There is some evidence that, in the dose range used clinically for ketamine-assisted psychotherapy, a local maxima exists for brain entropy that corresponds to maximal occipital alpha power suppression (147) without loss of consciousness.
3. The benefit of ketamine may rely not only on the biological effects of the drug but also on its impact on experience, as suggested by the fact that individuals who undergo treatment with ketamine at doses that induce anesthesia do not experience an antidepressant effect (28). Given the role of conscious experience, key factors for improving therapeutic outcomes will be set—the patient's intention and attitude—and setting—the physical environment of treatment (172). The physical setting should include a safe and comfortable environment free from interruption and conducive to relaxation—one that encourages individuals to fully attend to their internal experiences.
4. Dosage in ketamine treatment has often been kept below the level that produces a high entropy brain state in order to mitigate emergence side effects—visual, auditory, and sensate distortions experienced as disturbing by some patients in the process of awakening—or emerging—from ketamine sedation. Yet with proper management, these effects can take on a neutral or positive tone. In addition to a safe and comfortable setting, the addition of music is an important component of the therapeutic milieu that helps maintain a positive emotional tone (11, 23, 149, 172, 173). Therefore, a preselected program of music can be modulated to support emotional experiences while minimizing features that might introduce suggestions during ketamine infusion sessions. Music selections should be culturally appropriate. Individual selections within a program of music should vary in tempo and overall volume, so that the program has relatively quiet and tranquil selections as well as relatively active and dramatic sections. Instrumental music is generally preferable to music with lyrics in a language that the participant understands. Music sets will progress from music that is relaxing, proceed to pieces that are more active and emotionally evocative, and later move to quieter and more meditative selections. Therapists should be able to adjust the order of play as needed to fit the general mood and flow of the session, and a variety of music should be on hand in case a change to a different pre-recorded music set is needed. It is important for the attending professional to monitor individuals to ensure that the music is serving to support and deepen the process, rather than to distract from feelings or other experiences that may be emerging during the session.
5. Accurate measurement of ketamine effects will improve ketamine protocols by verifying effects associated with therapeutic outcomes.
  - Cortical activity in the motor cortex has been successfully employed as a measure of neuroplasticity and neurogenesis (126), time course of effects (24–48 h), and impacted brain regions. Hence, utilizing a paired associative stimulation (PAS) paradigm to assess neuroplasticity-induced changes pre and post ketamine treatment can help to confirm effective stimulation of neural plasticity.
  - Inter-hemispheric imbalance—specifically, hyperactive right-hemisphere and relatively hypoactive left-hemisphere—is associated with depression (174). The PAS paradigm provides a measurement of bi-hemispherical activity. Assessment of ketamine-induced cortical activity changes toward bi-hemispherical homeostasis, will help to confirm impacts associated with improved depression outcomes.
  - The presence of inflammation can be measured by means of readily available tests that assess the level of inflammatory cytokines present in a blood sample.
6. The inclusion of appropriately timed adjunctive psychotherapy sessions supports and enhances ketamine's mechanisms of action. It is thought that ketamine sets the stage and offers the experience necessary to forge new adaptive neural pathways in the brain, provided that psychedelic experiential integration occurs within the context of a supportive healing environment, and with the addition of timely psychotherapeutic interventions (11, 23, 149, 172, 173).
  - The therapist should be present during the ketamine experience, and for 45 min post induction to support patient safety, and to ensure ketamine experience integration with the patient's life experiences. Therapy session should also occur 24–48 h post ketamine induction to take advantage of peak neurogenesis, and neural plasticity, to enhance and maintain new neural pathways forged through psychedelic induction and reinforced through the dyadic therapeutic experience.

Despite numerous advances, research on ketamine treatment for TRD is still in relatively early stages. Evidence of new mechanisms of action and frames within which to organize an understanding of those mechanisms will continue to evolve as more research is advanced. Work with -R and S+ enantiomers of ketamine and norketamine may in time yield results demonstrating targeted efficacy for certain symptoms of depression or for certain populations. Innovative clinical protocols may provide treatment context that enhance ketamine's efficacy. Until more and better data are available, optimizing the use of racemic (R, S) ketamine by attending to ways in which its numerous mechanisms of action can best contribute to treatment may provide a more comprehensive response to the complex etiologies that underlie TRD.

At the same time, research is needed to clarify a number of areas related to ketamine therapy for TRD. The various efficacies of ketamine and norketamine enantiomers has already been mentioned; a major area of needed research is in translation of findings regarding ketamine's range of mechanisms of action from animal models to human subjects. In addition, research examining sex differences in ketamine response patters will deepen the understanding of ketamine's role in the complex relationships between inflammation and depression, in direct testing of the potential value of inducing entropic brain states to enhance ketamine's anti-depressant efficacy, and in searching for stronger evidence of correlations between the intensity of ketamine experiences and the extent and duration of anti-depressant outcomes. Work is also needed to build a more reliable understanding of ketamine's abuse liability and side effects in the clinical setting. In this research, the goal of clinical applications that account for and effectively deploy ketamine's multiple mechanisms in the most optimal way should remain at the forefront of discovery.

## Data Availability Statement

The original contributions presented in the study are included in the article/supplementary material, further inquiries can be directed to the corresponding authors.

## Author Contributions

S-AM: conceptualization and writing—original draft. GH: writing—review and editing, validation, and supervision. CC: writing—review and editing and supervision. KM: validation, software, review and editing, and supervision. All authors contributed to the article and approved the submitted version.

## Conflict of Interest

KM reports that he owns a patent referenced in this paper. The remaining authors declare that the research was conducted in the absence of any commercial or financial relationships that could be construed as a potential conflict of interest.

## Publisher's Note

All claims expressed in this article are solely those of the authors and do not necessarily represent those of their affiliated organizations, or those of the publisher, the editors and the reviewers. Any product that may be evaluated in this article, or claim that may be made by its manufacturer, is not guaranteed or endorsed by the publisher.
